# For Researchers on Obesity: Historical Review of Extra Body Weight Definitions

**DOI:** 10.1155/2016/2460285

**Published:** 2016-05-30

**Authors:** Marina Komaroff

**Affiliations:** Department of Epidemiology and Biostatistics, SUNY Downstate Medical Center, Brooklyn, NY 11203, USA

## Abstract

*Rationale.* The concept of obesity has been known since ancient world; however, the current standard definition of obesity was endorsed only about a decade ago. There is a need for researches to understand multiple approaches to defining obesity and how and why the standard definition was developed. The review will help to grasp the complexity of the problem and can lead to novel hypotheses in obesity research.* Objective.* This paper focuses on the objective to understand historical background on the development of “*reference *and* standard *tables” of weight as a platform for normal versus abnormal body weight definition.* Methods.* A systematic literature review was performed to chronologically summarize the definition of body weight from time of Hippocrates till the year of 2010.* Conclusion.* This paper presents the historical background on the development of “*reference *and* standard *tables” of weight as a platform for normal versus abnormal body weight definition. Knowledge of historical approaches to the concept of obesity can motivate researchers to find new hypotheses and utilize the appropriate obesity assessments to address their objectives.

## 1. Introduction

In 2001, “The Surgeon General's Call to Action to Prevent and Decrease Overweight and Obesity” set up the first objective as “to promote the recognition of overweight and obesity as major public health problem” in the United States [1]. The* Surgeon General's Call to Action *recognized that overweight and obesity have reached nationwide epidemic and underscored that the urgent goals are to prevent and treat the obesity and chronic diseases associated with obesity [1, 2].

In 2001, Food and Drug Administration and National Institutes of Health developed the* Healthy People 2010 *program that was committed to prevention and treatment of overweight and obesity to reduce chronic diseases associated with diet and weight [3]. In 2010 report,* Healthy People 2010 *summarized that the age-adjusted proportion of* healthy *(body mass index (BMI) < 25.0 kg/m^2^) adults (age ≥ 20 years) decreased from 42% in 1988–94 to 31% in 2005–08; and the proportion of* obese *(BMI ≥ 30.0 kg/m^2^) increased from 23% to 34% for the same period of time [3]. The experts concluded that the ten-year goal to prevent and treat the obesity was not met [3]. Instead, the nation was moving away from the objective to promote health and reduce chronic diseases associated with diet and weight [3]. This underscores the extent of the problem and urgency to seek a solution.

Clear understanding of the concept of obesity and its measurements should be the first step for successful research in obesity arena. The topic of obesity is extremely complex. Even though enormous research has been done to discover the causes of obesity, there is still no clear statement if obesity is exposure or outcome. For example, there is still a question if psychological depression causes obesity or obesity is a cause for psychological depression. This paper does not declare any perception on obesity and does not aim to cover causes and/or consequences of obesity. It aims to unfold all points of view on the obesity concept and definition through the chronological review of its development.

## 2. What Is Obesity

Hippocrates (460 BC–370 BC) described the health for human body as a balance of four humors (fluids): blood, black bile, yellow bile, and phlegm, and any deficiencies or extras were considered causes of diseases [4]. Obesity was defined as the surplus of humors [4]. For a big part of human history extra weight was considered as an indication of good health, as well as wealth and prosperity. Hippocrates was the first who realized that obesity leads to infertility and early mortality [5]. It took more than two thousand years to test his hypotheses scientifically and evaluate how “surplus of humors” relates to different health outcomes and early deaths.

The weight measurement and evaluation criteria are necessary to define overweight and obesity. In 1885, introduction of a penny scale first in Germany then in the United States allowed measuring the body weight to the nearest pound [6]. Since then a new era for weight assessments and search for “healthy” weight started. In the beginning of 20th century a low body weight was a concern because of threat of pneumonia and tuberculosis especially for young and underweight adults [7]. Healthy weight became the criteria for the individual's enrollment into the insurance policies [7–12]. The question of “ideal” weight and the acceptable level of deviation from “ideal” became the most important questions for insurance companies [7–12]. Overweight was defined as weight that exceeded the threshold from the* reference* value, where* reference *value was derived from the distribution of population. Based on observed association of body weight with mortality, the Metropolitan Life Insurance Company (MLIC) was able to develop the* standard *tables for “ideal” (MLIC 1942) [9, 10] and then “desirable” weight (MLIC 1959) [11, 12] and finally just “height to weight” tables (MLIC 1983) [13, 14]. Those* standard *tables became the platform for development of current definition for underweight, normal, overweight, and obese individuals based on the body mass index (BMI) cut-offs [15, 16].

## 3. Measurements of Obesity

One of the ways to assess overweight and obesity is to compare the individual's weight versus the* reference *value defined as the average population weight; and another way is to compare it with the weight* standards *that derived from the relationship between body weight and mortality or morbidity outcomes. The background of development* reference *or “average” tables is outlined in Section 3.1. The process of how tables of “average” weight were transformed into the “ideal” weight* standards *tables based on the lowest rate of mortality is described in Section 3.2. The historical background of development of both is summarized in Table 3.

### 3.1. “Normal Man” and the “Average” Weight Tables

Belgian astronomer and mathematician Adolphe Quetelet (1796–1874) was fascinated by the probability theory and passionately applied probability calculus into the physical characteristics of human body [19]. His idea was to demonstrate that normal Gaussian distribution can be applied to physical attributes of humans, and he was looking for the “norm” [19]. In 1831-32, Quetelet conducted what has been considered the first cross-sectional study of newborns and children based on their growth in height and weight [19]. Adolphe Quetelet's cross-sectional studies of human development revealed that weight grows as a cube of baby's height during the first year of life and then as a square of height until puberty and almost stops after the age of twenty-five [19]. In 1835, Quetelet published a book entitled “A Treatise on Man and the Development of His Aptitudes” with the conclusion that individual's weight increases as a function of the square of their height and introduced the anthropometric index as weight in kilograms divided by the square of the height in meters which is now known as Quetelet Index [19, years 1835 and 1842, English translation]. Adolphe Quetelet was the first who developed the table of the “average weight” at ages 20, 30, 40, 50, and 60 for a sample of Belgian men and women [7, 19].

In 1846, Hutchinson, a British surgeon, published a table of the “average” weights of 30-year-old men for each inch of height from 5 feet 1 inch to 6 feet at age of 30 based on statistics of 2,650 Englishmen [7, 20]. This table became the* reference *for English population and was used by the life insurance companies as a guide to evaluate applicants [7, 17].

In 1867, Fish published “*The Agent's Manual of Life Assurance*” by the Mutual Life Insurance Company of New York [17]. Table of height and weight (Table 1 [17, page 139]) adopted English* reference *for American people based on reviews from Minturn Post and Isaac L. Kip who were medical examiners for the Mutual Life Insurance Company of New York [17]. The* reference *table for American population “differs slightly” from the English ones set by Hutchinson and offered “what the best authorities regard as the most desirable proportion of the height of individuals to their weight” [17].

In 1889, the Actuarial Society of America and the Association of Life Insurance Medical Directors of America were founded which made possible a uniformed approach to the industry-wide height and weight tables [7]. In 1912, Medico-Actuarial Mortality Investigation published the statistics of height and weight of insured people and set up a goal “to provide an accurate* Standard *Table of Heights and Weights and to determine the influence of build on longevity” [7]. The whole process of development of* standard weight* tables with major milestones is summarized in Section 3.2 and Table 3.

### 3.2. Standard Tables of “Ideal” Weight

First tables of “ideal” weights were developed by Dublin and Lotha (1882–1969), a statistician and vice president of the Metropolitan Life Insurance Company (MLIC) [8]. The data from approximately four million MLIC policyholders from 1911 to 1935 were utilized [8]. The main criterion for “ideal” weight was longevity [8]. Dublin and Lotha grouped policyholders into categories by sex, height, and weights; but they could not fit the numbers into normal curve until they divided the studied population further into the three types of body frames: small, medium, and large [8]. From the prospective of insurance, the “bad weight” was considered to be 20–25%, with morbid obesity at 70–100% above the “ideal” weight for a given frame [8]. The “1942-43 Metropolitan Height and Weight Tables” were created based on Dublin and Lotha work and became the national* standards *for “ideal” body weight [9, 10]. Those tables were close representation of the longevity of American population but contained little information about trend and causes of mortality [9, 10].

In 1959, “*The Build and Blood Pressure Study*” was conducted by the Society of Actuaries with collaboration of 26 insurance companies to determine the mortality of insured persons according to variations in body build and blood pressure [12, 14]. The weights were derived from data of people with lowest mortality who were insured between 1935 and 1953 and followed to 1954 in the United States and Canada [12]. Measured heights and weights were of people wearing “indoor clothing and shoes” and included 20% of self-reported values [12]. “Frame size” for this study was not based on measurement of skeletal dimensions but arbitrarily divided the distribution of relative weights on the assumption that skeletal size was associated with a person's position in that distribution [12]. The association between body weight and mortality, particularly from cardiovascular diseases, was demonstrated, and MLIC 1959 weight tables were created, where “ideal weight” was replaced by the “desirable weight” [11].

In 1973, the Fogarty International Center Conference on Obesity recommended guidance for body weight based on the updates from the MLIC 1959 “desirable” weight tables [21]. The updates meant creation of “acceptable range” of weight for a particular height by the rule that for men and women for each inch of height set up the range of weight where the weight in the lower limit of the small frame and the weight in the upper limit of the large frame form the range of weights for the* standards* [21, 31]. The “acceptable range” for weight was converted into the suggested body mass index as 20.1–25.0 for men and 18.7–23.8 for women [21]. These tables were adopted in the report of obesity by Royal College of Physicians in 1983 [31].

In 1979, the second study of “The Build and Blood Pressure Study” produced the revised tables based on the mortality of insured people from 1950 to 1972 where “desirable” weights were higher than those in previous study [14]. Based on the data from new study, the MLIC 1959 tables for “desirable” weight were replaced by MLIC 1983 tables of “height and weight” [13].

Overweight and obesity are the nutrition-related disorders which are caused by the accumulation of extra fat as was stated by the Department of Health and Human Services (HHS) and the Department of Agriculture (USDA) which were responsible for issuing the “*Dietary Guidelines for Americans*” in 1980 and for updating the document every 5 years [32]. The goal of the document was to provide nutritional and dietary information and guidance for general public. In 1980, US Department of Agriculture and US Department of Health, Education and Welfare published the first edition of nutritional “*Dietary Guidelines for Americans*” and advised the public based on current knowledge of the relationship of diet to health and disease [18]. The guidance included a table with suggested body weight based on the tables recommended by Fogarty International Center Conference on Obesity with the adjustments for height and weight [18]. Adjustment for height was done as 1 or 2 inches (2.54 and 5.08 cm) for shoes and 10 lb–6 lb for clothes (Table 2) [18, 33, 34].

The National Institutes of Health Consensus Development Conference on the Health Implications of Obesity applied criteria for defining overweight, severe overweight, obesity, and severe obesity to the National Health and Nutrition Examination Survey 1976–80 (NHANES II) data [22]. “Overweight” was defined by a BMI (body mass index = weight in kilograms divided by weight in meters squared) ≥85th percentile, with “severe overweight” ≥95th percentile of 20-year-old to 29-year-old men and women [22]. “Obesity” and “severe obesity” were defined using the same criteria: only the sum of the triceps and subscapular skinfold thicknesses instead of the body mass index [22]. The numbers came to the criteria of defining “overweight” as BMI ≥27.8 kg/m^2^ for men and BMI ≥27.3 for women [22]. These criteria matched with 20% or more above the “desirable” weight in the MLIC 1983 tables (from midpoint of the range for a medium-build person) [22]. These definitions became widely accepted as the* standards* [22]. Nevertheless, numbers for ranges of acceptable “normal” BMI were different from those based on the version of* standard *tables, for example, for MLIC 1959, 26.4 (men) and 28.5 (women), and for MLIC 1983, 27.2 (men) and 26.9 (women) [23]. Moreover, the recommended requirement differed from the guidelines set up by Fogarty Center and the Royal College of Physicians [21, 35]. Such discrepancies in the guidance pointed out the technical problems of weight to height tables: the discrepancies of measurements (shoes, clothes, and approximately 20% self-reported data in MLIC 1959 and 10% in MLIC 1983) and population selection [35]. It brought up an urgent need to standardize definition of overweight and obesity and the search for weight to height index of body weight started.

In 1985, the Department of Health and Human Services (HHS) and the Department of Agriculture (USDA) issued the second edition of “*Dietary Guidelines for Americans*” [24]. That document used the “desirable” weight to height tables from MLIC 1959 and translated the numbers into overweight level of BMI for men ~25-26 and for women ~24-25 [24, 34]. In the 1990 third edition, tables for men and women were combined into one and presented by two age groups where “unhealthy” weight was translated into BMI ≥ 25 for ages 19–34 and ≥27 for ages ≥35 years [25, 34].

In 1990, the “National Nutrition Monitoring and Related Research Act of 1990,” Public Law 101–445, Title III, Section301, stated that a report entitled “*Dietary Guidelines for Americans*” shall contain nutritional and dietary information and guidelines for the general public and be published at least every five years [28]. Each such report “shall be promoted by each Federal agency in carrying out any Federal food, nutrition, or health program” [28]. Basically, the law stated that the definition of healthy weight or overweight from the Dietary Guidelines for Americans should be used by the constituent Federal agencies [28].

In Geneva, in 1995, WHO Expert Committee on Physical Status published technical report:* The Use and Interpretation of Anthropometry* [15]. It was acknowledged that the basic anthropometry measurements of human body are weight and height [15]. It was recommended to combine two basic measurements: weight and height into the body mass index (weight/height^2^) or ponderal index (weight/height^3^) for adults; and three anthropometric indexes with consideration of age were described for children: weight for height, height for age, and weight for age [15]. The WHO Expert Committee on Physical Status proposed to classify different levels of BMI with cut-off points of 25, 30, and 40 based on “arbitrary method of association between BMI and mortality” [15]. WHO Expert Committee on Physical Status referenced the meta-analysis performed by Troiano et al. which included 17 studies with 37 substudies with 350,000 men and >38000 deaths plus six studies with 12 substudies with 250,000 women and 13700 deaths and presented the U-shape mortality rates that sharply increased when BMI <18.5 and >30.0 kg/m^2^ with the acceptable BMI range as 18.5–25.00 [15, page 322]. The WHO experts underscored that the cut-offs were chosen arbitrarily based on the “visual inspection of the relationship between BMI and mortality” [15] curve: for example, “the cut-off of 30 is based on the point of flexion of the curve” [15]. The authors suggested that this method should be revised in the future in terms of BMI association with health risk [15].

In 1995, the next step was taken to combine not just both sexes but also age groups where “healthy” weight was translated in BMI ≥25 for all adults in the fourth edition of “*Dietary Guidelines for Americans*” [27]. The report used WHO criteria for publishing healthy weight ranges for men and women by each inch of height [27]. The edition was very important because it led to publishing the prevalence and trends on overweight (BMI ≥ 25), preobese (BMI: 25.0–29.9), class 1 (BMI: 30.0–34.9), class 2 (BMI: 35.0–39.9), and class 3 (BMI ≥ 40.0) obese in the United States data from 1960 to 1994 (NHANES I, II, and III) for first time to facilitate comparison with international data [2].

*Clinical Guidelines on the Identification, Evaluation, and Treatment of Overweight and Obesity in Adults (NIH, September 1998)* defined overweight as a body mass index (BMI) of 25 to 29.9 kg/m^2^ and obesity as a BMI of ≥30 kg/m^2^ [2]. The panel of experts came to this classification based on evidence from approximately 394 randomized controlled trials (RCTs) and multiple observational studies about BMI and risk of morbidity and mortality [2]. The classification matched with one suggested by WHO [2, 15].

In 2000, World Health Organization (WHO) defined overweight and obesity as “the disease in which excess body fat has accumulated to such an extent that health may be adversely affected” [26] and underscored that the practical definition of obesity is based on the level of body mass index (BMI) [15, 26]. The most recent version of international classification for adults developed by WHO is presented in Table 4 [15, 26].

BMI has never been a perfect index for children because it correlates with height. In the United States, Centers for Disease Control and Prevention (CDC) 2000 used five nationally representative surveys to develop BMI charts for children in the United States: the National Health Examination Surveys (NHES) II and III in the 1960s, the National Health and Nutrition Examination Surveys (NHANES) I and II in the 1970s, and NHANES III for the period of 1988–1994. Those charts included gender-specific BMI by single month of age curves of growth. Currently they are used in the United States for children who are 2–19 years old to define “overweight” as BMI ≥95th percentile or “at risk for overweight” if BMI is between 85th and 95th percentiles [26, 36].

International surveys of six large and nationally representative cross-sectional growth studies from Brazil, Great Britain, Hong Kong, Netherlands, Singapore, and the United States were used by International Obesity Task Force (IOTF) to establish the cut-off points for overweight and obese among children and adolescents [37]. In summary, participants of IOTF agreed to link childhood overweight and obesity to the cut-offs for adults. Based on each of the surveys, percentile curves were drawn in such a way that they passed through BMI cut-off points of 25 and 30 kg/m^2^ at age of 18 years, and the resulting curves were averaged by age and gender to provide cut-off points for 2 through 18 years of age [37]. This way less arbitrary and more internationally based cut-offs were proposed [37].

In the current edition of* Dietary Guidelines for Americans, 2010*, the BMI cut-offs are consistent with internationally recommended [16]. By the Public Law 101–445, Title III, Section301, all Federal agencies have made the transition to define overweight and obesity as a BMI consistent with recommendations in the current edition of* Dietary Guidelines for Americans* [16, 28]. The table of weight category based on BMI level from* Dietary Guidelines for Americans, 2010*, is presented in Table 5 [16].

## 4. Summary

There is nationwide and global epidemic of obesity. As the first step to conquer a problem, the concept of obesity should be clearly understood through the historical process that led to the worldwide accepted standard definition. This paper presents the history of developing current standard definition of overweight and obesity. Understanding the roots will lead to the successful research on obesity with a goal to defeat national and global epidemic.

## Figures and Tables

**Table 1:** Table of height and weight [17, page 139].

Height		Weight (pounds)
Feet	Inches	
5	1	120
5	2	125
5	3	130
5	4	135
5	5	140
5	6	143
5	7	145
5	8	148
5	9	155
5	10	160
5	11	165
6	∞	170

**Table 2:** Range of acceptable body weight [18].

Height (feet-inches)	Men (pounds)	Women (pounds)
4′10′′		92–119
4′11′′		96–129
5′0′′		96–125
5′1′′		99–128
5′2′′	112–141	102–131
5′3′′	115–144	105–134
5′4′′	118–148	108–138
5′5′′	121–152	111–142
5′6′′	124–156	114–146
5′7′′	128–161	118–150
5′8′′	132–166	122–154
5′9′′	136–170	126–150
5′10′′	140–174	130–163
5′11′′	144–179	134–168
6′0′′	148–184	138–173
6′1′′	152–189	
6′2′′	156–194	
6′3′′	160–199	
6′4′′	164–204	

Note: height without shoes and weight without clothes.

**Table 3:** Development of weight standard tables.

Year [ref.]	Author	Criteria^*∗*^	Population	Screening	Development	Milestone/outcome
1842 [19]	Quetelet	*Tables*: weight to height by age group: 20, 30, 40, 50, and 60	Belgian men and women	With clothes and shoes (as is)	*Developed a table of the average weight by ages for a sample of Belgian men and women*	“*Average *weight” *Belgian*

1846 [20]	Hutchinson	*Tables*: weight to height for 30 y.o. Englishmen from 5′1′′ to 6 feet	5 feet 1 inch to 6 feet, 30 y.o. 2,650 Englishmen		Average weights of 30 y.o. men for each inch of height from 5′1′′ to 6 feet	“*Average* weight” * English*

1867 [17]	Fish	*Tables*: weight to height for 30 y.o. American men from 5′1′′ to 6 feet	American	Medical Review of English Tables (from John Hutchinson)	*The Agent's Manual of Life Assurance*	“*Average* weight” American

1912 [7]	The Association of Life Insurance Medical Directors and the Actuarial Society of America	*Tables*: based on *rate of mortality*: weight by height tables for men and women *by age*	Mortality for insured population	Seasonal consideration to have the same clothes and shoes	*Report of The Joint Committee onThe Medico-Actuarial MortalityInvestigation* “Average weight may not have the lowest mortality”—what is ideal weight?	“*Ideal *weight” based on *longevity*

1937 [8]	Dublin and Lotha (1882–1969), Metropolitan Life Insurance Company frame [8], 1937	*Tables*: weight to height by sex and *body frame*	~4 million people insured by MetLife 1911–1935	Height, with street shoes Weight, only indoor clothes	Dublin divided the average weights into three types of body frames: small, medium, and large From the prospective of insurance, the bad weight was considered to be 20–25%, with morbid obesity at 70–100% above the “ideal”	“*Ideal *weight” (based on lowest mortality for insured population)

1942-3 [9, 10]	Metropolitan Life Insurance Company (MLIC)	*Tables*: weight by height by sex and body frame Longevity criteria, weight maintained at the same level as 25 y.o. with same height and body frame	Proposed tables for “ideal weight” for men and women >25 y.o. by frame size	Height, with street shoes Weight, only indoor clothes	*National Standards for Weight by sex and body frame MLIC 1942-43* Obesity, 20% above ideal weight	“*Ideal* weight”

1959 [11, 12]	The Build and Blood Pressure Study	*Tables: *weight by height by sex and body frame	US and Canada-insured (from 26 life insurance companies) people 1935–1954 20% self-reported	Street shoes and indoor clothes	*Revised MLIC 1959 tables* *Standard Tables based on association with mortality *(not morbidity) distribution of weight to height association with minimum mortality (lowest death rate) Obesity, 20% above ideal weight	“*Desirable* weight”

1975 [21]	Fogarty Center Conference on Obesity	Range of acceptable weight Suggested range BMI: 20.1–25.0 for men 18.7–23.8 for women	Based on MLIC 1959	Adjusted street shoes and indoor clothes	*Guidance for body weight*	*Range of acceptable weight*

1979 [13, 14]	The Build and Blood Pressure Study, second study	*Tables*: weight by height by sex and body frame	Insured people 1950–1972 10% self-reported	Street shoes and indoor clothes	*Revised MLIC 1983 tables*	“*Height to weight tables*”

1980 [18]	US Department of Agriculture and US Department of Health, Education and Welfare	Adjusted range of acceptable weight of age and height specific	MLIC 59	Adjusted street shoes and indoor clothes	*Dietary Guidance for Americans (1st edition)*	*Range of acceptable weight* from “desirable” weight tables

1985 [22, 23]	National Institute of Health	“Overweight”: BMI ≥ 85th percentile, “severe overweight”: BMI ≥ 95th percentile of 20-year-old to 29-year-old men and women. “Obesity” and “severe obesity” same for the sum of the triceps and subscapular skinfold thicknesses	NHANES II 1976–80 Men and women 20–29 years old	Criteria matched for range of acceptable weight: not ≥20% of desirable weight for midpoint of medium-build person MLIC, 83.	*Guidance for body weight* Overweight-obesity BMI: ≥27.8 for men ≥27.3 for women *Suggested range BMI for males and females*: 26.4–28.5 *(MLIC 59) *27.2–26.9 *(MLIC 83)*	*Range of acceptable weight*

1985 [24]	US Department of Agriculture and US Department of Health, Education and Welfare	Overweight translated to BMI for men ~25-26 and for women ~24-25	MLIC 59		*Dietary Guidance for Americans (2nd edition)*	*Desirable weight*

1990 [25]	US Department of Agriculture and US Department of Health, Education and Welfare	BMI ≥ 25 for ages 19–34 and ≥27 for ages ≥35 years		All adults (both sexes combined) by 2 age groups	*Dietary Guidance for Americans (3rd edition)* translated in BMI ≥25 for ages 19–34 and ≥27 for ages ≥35 years	*Unhealthy weight*

1995 [15] 2000 [26]	World Health Organization (WHO)	Mortality curve	International data	Mortality curves for BMI selection	*International Classification of Weight*	*International Classification of Weight based on BMI level*

1995 [27]	US Department of Agriculture and US Department of Health, Education and Welfare	BMI ≥ 25 for all ages	All adults (both sexes combined) for all ages	No shoes, no clothes	*Dietary Guidance for Americans (4th edition)*	*Healthy weight*

1998 [2]	National Institute of Health (NIH) and National Heart, Lung, and Blood Institute (NHLBI)	Relation of BMI and risk of morbidity and mortality	RCT and epidemiological studies		*Clinical Guidelines on the Identification, Evaluation, and Treatment of Overweight and Obesity in Adults*	*Assessment and treatment management of overweight and obesity*

2010 [16]	US Department of Agriculture and US Department of Health, Education and Welfare	WHO International Classification of Weight based on BMI level was adopted for US population	USA	Matches with WHO and NIH and NHLBI criteria	*Dietary Guidance for Americans *(*7th edition*)^*∗∗*^	*BMI classification matches with WHO and NIH and NHLBI, 1998*

^*∗*^Criteria for table generation.

^*∗∗*^Next edition is expected in 2015.

“National Nutrition Monitoring and Related Research Act of 1990”—*Public Law 101–445, Title III, Section  301,* stated that a report entitled “*Dietary Guidelines for Americans*” shall contain nutritional and dietary information and guidelines for the general public and “shall be promoted by each Federal agency in carrying out any Federal food, nutrition, or health program” [28].

**Table 4:** International classification of adults based on body mass index.

Classification	BMI (kg/m^2^)	
	Cut-off points	Additional cut-off points
*Underweight*	*<18.50*	*<18.5*
Severe thinness	<16.00	<16.0
Moderate thinness	16.00–16.99	16.00–16.99
Mild thinness	17.00–18.49	17.00–18.49
*Normal*	*18.50–24.99*	*18.50–22.99*
		*23.00–24.99*
*Overweight*	*≥25.00*	*≥25.00*
Preobese	25.00–29.99	25.00–27.49
		27.50–29.99
*Obese*	*≥30.00*	*≥30.00*
Class I	30.00–34.99	30.00–32.49
		32.50–34.99
Class II	35.00–39.99	35.00–37.49
		37.50–39.99
Class III	≥40.00	≥40.00

Source: from WHO, 1995, WHO, 2000, and WHO, 2004 [15, 26, 29, 30].

**Table 5:** Overweight and obese children and adolescents [16].

Category	Children and adolescents (BMI for age percentile range^*∗*^)	Adults (BMI)
Underweight	Less than the 5th percentile	Less than 18.5 kg/m^2^
Healthy weight	5th percentile to less than the 85th percentile	18.5 to 24.9 kg/m^2^
Overweight	85th percentile to less than the 95th percentile	25.0 to 29.9 kg/m^2^
Obese	Equal to or greater than the 95th percentile	30.0 kg/m^2^ or greater

^*∗*^Growth charts are available at http://www.cdc.gov/growthcharts/.
